# HLA-G neo-expression modifies genetic programs governing tumor cell lines

**DOI:** 10.1007/s00262-024-03768-5

**Published:** 2024-10-03

**Authors:** Diana Tronik-Le Roux, Marina Daouya, Isabelle Poras, François Desgrandchamps, Edgardo D. Carosella

**Affiliations:** 1 CEA Commissariat À L’Énergie Atomique Et Aux Énergies Alternatives/Atomic Energy and Alternative Energies Agency, HIRD Hematology and Immunology Research Division, Saint-Louis Hospital, 1 Avenue Claude Vellefaux, 75010 Paris, France; 2 UMRS Unité Mixte de Recherche Et de Service 976HIPI, Human Immunology Pathophysiology Immunotherapie Unit, IRSL Institut de Recherche Saint Louis, University of Paris, Saint-Louis Hospital, 1 Avenue Claude Vellefaux, 75010 Paris, France; 3 Department of Urology, Saint-Louis Hospital, 1 Avenue Claude Vellefaux, 75010 Paris, France

**Keywords:** HLA-G, Immune checkpoints, ccRCC, Transcriptome analysis, Cancer markers

## Abstract

The development of immunotherapies has proved to be clinically encouraging to re-establish the immune function modified by the expression of immune inhibitory molecules in tumors. However, there are still patients with poor survival rates following treatment. The elucidation of molecular mechanisms triggered by the neo-expression of particular IC in tumors would constitute a major step toward better understanding tumor evolution and would help to design future clinical protocols. To this end, we investigate the modifications triggered by the neo-expression of the immune checkpoints HLA-G in ccRCC tumor cells. We demonstrate, for the first time, that HLA-G modifies key genes implicated mainly in tumor development, angiogenesis, calcium flow and mitochondria dynamics. The involvement of HLA-G on the expression of genes belonging to these pathways such as ADAM-12, NCAM1 and NRP1 was confirmed by the CRISPR/Cas9-mediated edition of HLA-G. The data reveal multifaceted roles of HLA-G in tumor cells which are far beyond the well-known function of HLA-G in the immune anti-tumor response. This warrants further investigation of HLA-G and these new partners in tumors of different origin so as to propose future new treatments to improve health patient’s outcome.

**Supplementary Information:**

The online version contains supplementary material available at 10.1007/s00262-024-03768-5.

## Introduction

Nascent transformed cells begin to acquire the expression of immune inhibitory molecules, termed immune checkpoints (IC), which promote the generation of immune-tolerant microenvironments allowing tumor cells to escape the immune anti-tumor response. Despite the advances made in immunotherapy treatments, many questions remain. These might be answered by the systematic investigation of the molecular mechanisms underlying the action of particular IC in cancer cells and, in this way, pinpointing potential new targets or synergistic combinations that might improve therapeutic protocols.

Clear cell renal cell carcinoma (ccRCC) is the most common epithelial tumor of the kidney that occurs in adults, accounting for approximately 70–80% of renal cancers. ccRCC is characterized by malignant epithelial cells with clear cytoplasm and a compact-alveolar or acinar growth pattern interspersed with intricate, arborizing vasculature. Prognosis is generally poor due to insufficient early warning signs. Hallmarks of ccRCC are high vascularization, immunogenic properties, mitochondrial dysfunction and disturbed ion transport channels [1]. When tumors cannot be sufficiently oxygenated, hypoxia occurs and triggers the upregulation of several genes, including VEGF and a variety of IC. The persistent hypersecretion of these pro-angiogenic factors within the tumor leads to the disorganization of nascent vessels, preventing T cell infiltration [2] overall driving a shift toward an immunotolerant environment. Therefore, the first-line treatments have been based on the administration of anti-angiogenic drugs such as the vascular endothelial growth factor (VEGF) neutralizing antibody termed Bevacizumab [3]. Despite proven benefits, these drugs offer only a modest survival benefit. In consequence, novel strategies might be developed to improve the efficacy of conventional anti-angiogenic treatments. One option could be blocking immune checkpoints.

The non-classical class I human leukocyte antigen G (HLA-G) was first described for having a major role in feto-maternal tolerance and tissue transplantation [4]. Since then, HLA-G was found to be involved in a variety of processes such as viral infection, autoimmune diseases and defined as an immune checkpoint found in most tumors. In particular, high incidence of HLA-G expression has been reported in ccRCC [5–7]. Consequently, HLA-G constitutes a challenging target which, to be used, needs extended research efforts to identify the molecular basis of its action.

Here, by using a ccRCC cellular model, we demonstrate, for the first time, that HLA-G modifies the expression of genes related to tumor development, tumor angiogenesis, mitochondria metabolism and ion channels, in the absence of its known receptors ILT2, ILT4 or KIR2DL4. The edition of HLA-G using the CRISPR/Cas9 method further confirmed the involvement of HLA-G in these processes. Altogether, the data provide a comprehensive survey of the complex gene networks that are altered by the neo-expression of HLA-G in tumors. Undoubtedly, this would be useful to propose new treatment strategies and drug targets to improve health patient’s outcome.

## Materials and methods

### Cell lines

The RCC7 cell line is derived from a clear cell renal cell carcinoma patient, collected with the prior consent of the donor and anonymized, kindly provided by Dr. Anne Caignard [8]. The RCC7 line does not express HLA-G, but it was previously transduced with lentivirus containing HLA-G1 cDNA, generating a stable cell line expressing high levels of HLA-G1 isoform (RCC7/HLA-G1). The GFP cDNA was also transduced in RCC7 cells (pWPXL, Addgene plasmid # 12257). The M8 melanoma cell line into which we transduced or not the HLA-G cDNA was previously described [9].

RCC7 and M8 cells were grown in DMEM (Gibco™ Thermo Fisher Scientific™, Waltham, MA, U.S.) or RPMI (Merck KGaA, Darmstadt, Germany), respectively, supplemented with 10% heat‐inactivated fetal calf serum (Merck), 1% amphotericin B 250 µg/ml (Gibco™) and 1% gentamicin 10 mg/l (Gibco™). Cultures were maintained at 37 °C and 5% CO_2_ and routinely tested for mycoplasma contamination by using the LookOut® Mycoplasma PCR Detection Kit (Merck). Cells were regularly dissociated using PBS-EDTA, 2 mM (Merck).

**Fig. 1:**
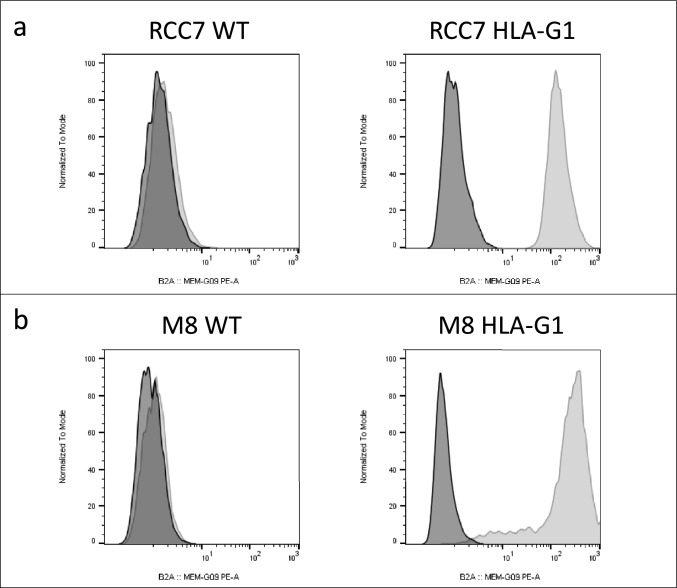
Flow cytometry analysis of the expression of HLA-G in RCC7 (**a**) and M8 cells (**b**) using the HLA-G-specific antibody MEM-G/9-PE. Isotype: dark peaks; HLA-G: light gray peaks

The verification of the expression of HLA-G in RCC7 (Fig. 1a) and M8 (Fig. 1b) cells in WT and HLA-G-expressing cells was performed by flow cytometry. Plots are shown in Fig. 1.

### Antibodies and flow cytometry analysis

For flow cytometry analysis, approximately 10^4^ cells were analyzed for each experiment. The following antibodies were used for cell surface staining: MEM-G/9-PE (anti-HLA-G1) from Exbio (Praha, a.s.); ILT2-PE (Clone: HP-F1) and ILT4-PE from eBioscience (San Diego, CA, U.S.); CD147-PE, SDC2-APC; CD56-PE, CD56-FITC and HVEM-PE from Miltenyi Biotec (Bergisch Gladbach, North Rhine-Westphalia, Germany); KIR2DL4-PE; NRP1-PE from BioLegend (San Diego, CA, U.S.); CD160 from Abcam (Cambridge, U.K.) and CD160-PE from Beckman Coulter (Brea, CA, U.S.). Isotyping, primary and secondary antibodies staining were performed according to the manufacturers’ instructions. Acquisition was performed on a MACSQuant® 10 flow cytometer (Miltenyi Biotec); analysis was performed using the MACSQuantify™ software (Miltenyi Biotec) and FlowJo™ software.

### RNA extraction

Total RNA was isolated using RNeasy Kit (QIAGEN GmbH, Hilden, Germany) according to the manufacturer’s instructions and treated with DNase (RNase-free DNase Set, QIAGEN). The RNA purity and concentration were assessed using Nanodrop spectrophotometer (Nanodrop, Thermo Fisher Scientific™, Waltham, MA, U.S.). Samples were stored at − 80 °C before use.

### Microarray procedure

Biotinylated cDNA (10 μg) prepared from total RNA samples prepared from ccRCC-expressing HLA-G and the control gene GFP were hybridized on Affymetrix® ClariomsHUman chips. Microarrays were scanned using GeneChip™ Scanner 3000 7G (Affymetrix Inc., Santa Clara, CA, U.S.) and were processed with GeneChip™ Operating System to generate the CEL file expression data. All analyses were conducted according to the manufacturer’s instructions at the Genomics core facility of the Cochin Institute, Paris, France.

### Statistical analysis of gene expression data

Transcriptomic analysis was performed in R (version 4.1.2). Expression data were normalized with RMA method implemented in oligo R package (version 1.58.0). For features filtering, we used the function nsFilter in genefilter R package (version 1.76.0), which calculates the variance of each probe across the samples and identifies the ones with low variance. Probes with variance less than the 0.75 quantile were filtered out. Differential analysis was performed by applying an empirical Bayes statistic to a linear model fit (functions lmFit and eBayes in limma R package, version 3.50.0). *p* values < 5% were considered significant.

### Functional and pathway enrichment analysis of DEGs

To analyze the identified DEGs at the functional level, we used enrichR package. We looked for functional enrichment in the following databases: WikiPathway 2021 Human, MSigDB Hallmark 2020, KEGG 2021 Human, Reactome 2016, GO Biological Process 2021, GO Cellular Component 2021 and GO Molecular Function 2021. We selected the top 10 terms based on the adjusted *p* value of the enrichment test.

### Real-time quantitative RT-PCR

For cDNA synthesis, 1 µg of total RNA was retro-transcribed using the High-Capacity cDNA Reverse Transcription Kit (Applied Biosystems™, Foster City, CA, U.S.) following the manufacturer's instructions. The reverse transcription reaction was carried out with a Mastercycler® pro vapo.protect™ (Eppendorf®, Montesson, France). The real-time quantitative PCR assays were performed using a Power SYBR® Green PCR Master Mix (Applied Biosystems™) in an ABI Prism™ 7000 SDS (Applied Biosystems™). Data were normalized according to the expression of the housekeeping gene beta-actin, and the relative expression of each mRNA was calculated with the 2^−∆∆Ct^ method.

### CRISPR/Cas9-HLA-G-edited RCC7/HLA-G1 cells

CRISPR/Cas9-HLA-G knockout was generated by viral transduction of a sgRNA complementary to a region just upstream of the ATG located in exon 2 which is the main translation start codon used by most of the HLA-G isoforms [10].

### tBHQ blocking assay

10^6^ of RCC7 cells (WT and HLA-G1) per well were settled in a Falcon® 6-Well Flat-Bottom Plate in 2 ml of DMEM supplemented with 10% FSC and 200mM L-glutamine, for 2 h, with different concentrations of tBHQ (Sigma-Aldrich, Merck) [0, 2, 5, 10, 15 and 20 µM]. Then cells were taken up in RLT buffer (QIAGEN) for subsequent RNA analysis.

### Calcium inhibition assay

The Fluo-4 Assay™ Kit Calcium (Abcam) was used for the calcium inhibition assay as indicated by the manufacturer. Briefly, 25.000 RCC7 cells (WT and HLA-G1) were settled per well in a Falcon® 96-Well Black Wall Flat-Bottom Plate in 100 µl of DMEM supplemented with 10% FSC and 200mM L-glutamine for 24 h. Prior to the calcium assay, cells were incubated in fresh medium in the presence or absence of serum. Extemporaneously, histamine dihydrochloride (Merck) was added at a final concentration of 1 µM. Acquisition was performed on a CLARIOstar^plus^ (BMG LABTECH, Offenburg, Germany), excitation: 494 nm/emission 516 nm.

### NRP1 inhibition assay

10^6^ of RCC7 cells (WT and HLA-G1) per well were settled in a Falcon® 6-Well Flat-Bottom Plate in duplicates (one for the flow cytometry staining and one for the RNA extraction) in 2 ml of DMEM supplemented with 10% FSC and 200mM L-glutamine for 30 min with an increasing concentration of EG01377 (from 0 to 2 µM).

After 30 min, cells were collected. One half was taken up in RLT buffer (QIAGEN) for subsequent RNA analysis. The other half was stained for flow cytometry analysis with an anti-HLA-G antibody (MEM-G/9-PE,s EXBIO Praha, a.s.).

## Results

### Identification of novel genes associated with HLA-G expression in RCC7 cells

To study potential gene modification triggered by the neo-expression of HLA-G in tumor cells, we compared gene expression profiles of RCC7 cells expressing or not HLA-G. To this end, RNA was extracted from RCC7 cells carrying the lentivirus PWXL encoding either the HLA-G or the GFP cDNA. Following retro-transcription of these RNAs, samples were subject to DNA microarray profiling to perform pairwise comparisons using Human Clariom Affymetrix GeneChip as described in materials and methods.

**Fig. 2:**
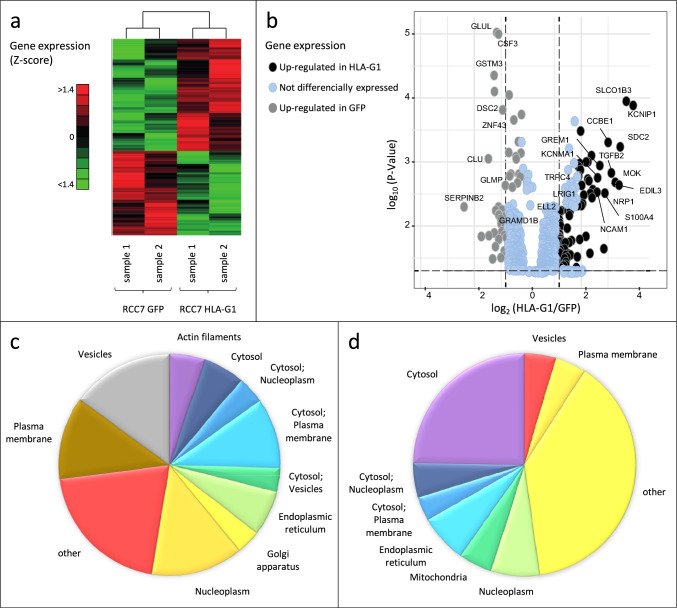
Gene expression modifications triggered by HLA-G. **a** Heat map plot showing the most differentially expressed genes between HLA-G and GFP samples. **b** Volcano plot showing the most up- and downregulated genes (right). **c**, **d** Pie chart representing the distribution of the subcellular localization of the 77 upregulated and the 56 downregulated genes found in HLA-G versus GFP-expressing cells, respectively

Using a *t* test with *p *value corrected by the Benjamini–Hochberg algorithm, we found 133 genes that were differentially expressed with a *p *value significance level of 0.05 (Table 1). These were distributed in a relatively balanced manner between over- and under-expressed genes, 77 and 56 genes, respectively. The most differentially expressed genes between the HLA-G and GFP conditions are represented in a Heat map plot showing their relative abundance. To schematize the level of gene regulation, genes were grouped by a hierarchical clustering using Pearson metric and Ward distance and a Volcano plot (Fig. 2a and 2b, respectively).

**Table 1:** List of the 133 DEGs identified from the profile datasets, including 77 upregulated genes and 56 downregulated genes in RCC7 cells expressing HLA-G, compared to RCC7 cells expressing GFP

Symbol	Gene name	Symbol	Gene name
*UP in RCC7-HLA-G1*			
SDC2	Syndecan 2	PTPRE	Protein tyrosine phosphatase, receptor type, e
C1orf21	Chromosome 1 open reading frame 21	PANK2	Pantothenate kinase 2
WDFY2	WD repeat and FYVE domain-containing 2	LOXL1	Lysyl oxidase-like 1
NTM	Neurotrimin	HOXC10	Homeobox c10
GPR180	G protein-coupled receptor 180	ISYNA1	Inositol-3-phosphate synthase 1
TGFBR3L	Transforming growth factor beta receptor III like	TSPAN2	Tetraspanin 2
HLA-G	Major histocompatibility complex, class I, G	LSAMP	Limbic system-associated membrane protein
TGFB2	Transforming growth factor beta 2	NALCN	Sodium leak channel, non-selective
CYB5A	Cytochrome B5 type A (microsomal)	ATP8B1	ATPase, aminophospholipid transporter, class i, type 8B, member 1
KCNMA1	Potassium channel, calcium-activated large conductance, subfamily M A, member 1	EDNRA	Endothelin receptor type A
DTNA	Dystrobrevin, alpha	ADGRG6	Adhesion G protein-coupled receptor G6
LAYN	Layilin	MYLK	Myosin light chain kinase
MOK	MOK protein kinase	RASSF8	Ras association (ralgds/af-6) domain family (n-ter) member 8
EDIL3	EGF-like repeats and discoidin I-like domains 3	ANKRD1	Ankyrin repeat domain 1 (cardiac muscle)
ADK	Adenosine kinase	ITGA2	Integrin, alpha 2 (cd49b, alpha 2 subunit of vla-2 receptor)
TRPC4	Transient receptor potential cation channel, subfamily C, member 4	FRG2	FSHD region gene 2
S100A4	S100 calcium binding protein A4	SPARC	Secreted protein, acidic, cysteine-rich (osteonectin)
NRP1	Neuropilin-1	DCLK1	Doublecortin-like kinase 1
VCAN	Versican	LIPA	Lipase a, lysosomal acid, cholesterol esterase
PDLIM1	PDZ and LIM domain 1	HADH	Hydroxyacyl-CoA dehydrogenase
LRIG1	Leucine-rich repeats and immunoglobulin-like domains 1	FUT8	Fucosyltransferase 8 (alpha (1,6) fucosyltransferase)
GRK5	G protein-coupled receptor kinase 5	FRG2B	FSHD region gene 2 family, member B
PLPP4	Phospholipid phosphatase 4	HCFC1R1	Host cell factor C1 regulator 1 (XPO1-dependent)
GREM1	Gremlin 1, DAN family BMP antagonist	MATN2	Matrilin 2
INHBA	Inhibin beta A	OTUD1	OTU deubiquitinase 1
SMOC1	SPARC-related modular calcium binding 1	TSPAN1	Tetraspanin 1
MYOF	Myoferlin	LOX	Lysyl oxidase
GABBR2	Gamma-aminobutyric acid (GABA) B receptor, 2	TGFBI	Transforming growth factor, beta-induced, 68kDa
NCAM1	Neural cell adhesion molecule 1	PRKCA	Protein kinase C, alpha
APBB1IP	Amyloid beta (A4) precursor protein-binding, family B, member 1 interacting protein	CPM	Carboxypeptidase M
ELL2	Elongation factor, RNA polymerase II, 2	SNAPC1	Small nuclear RNA activating complex polypeptide 1
ALG9	ALG9, alpha-1,2-mannosyltransferase	SNRPN	Small nuclear ribonucleoprotein polypeptide N
MTSS1	Metastasis suppressor 1	CDH6	Cadherin 6, type 2, K-cadherin (fetal kidney)
LRIG1	Leucine-rich repeats and immunoglobulin-like domains 1	CCDC186	Coiled-coil domain-containing 186
CHRDL1	Chordin-like 1	MYO1D	Myosin ID
ALOX5AP	Arachidonate 5-lipoxygenase-activating protein	ADAM-12	Adam metallopeptidase domain 12
*UP in RCC7 GFP*			
Symbol	Gene name	Symbol	Gene name
GLUL	Glutamate-ammonia ligase	KCNIP1	Kv channel interacting protein 1
CSF3	Colony stimulating factor 3	ENDOD1	Endonuclease domain-containing 1
GSTM3	Glutathione-S-transferase mu 3 (brain)	CCBE1	Collagen and calcium binding EGF domains 1
DSC2	Desmocollin 2	SH2D4A	SH2 domain-containing 4a
GYPC	Memczak2013 ANTISENSE, coding, INTERNAL, UTR3 best transcript NM016815	PPP4R4	Protein phosphatase 4, regulatory subunit 4
ZNF43	Zinc finger protein 43	ZNF189	Zinc finger protein 189
SORBS2	Sorbin and SH3 domain-containing 2	EEF1A2	Eukaryotic translation elongation factor 1 alpha 2
SP140	SP140 nuclear body protein	BHMT2	Betaine–homocysteine S-methyltransferase 2
ANKEF1	Ankyrin repeat and EF-hand domain-containing 1	ALX1	ALX homeobox 1
NSUN3	NOP2/Sun domain family, member 3	TSPAN18	Tetraspanin 18
FKTN	Fukutin	SPINK13	Serine peptidase inhibitor, kazal type 13 (putative)
KIAA1324L	KIAA1324-like	UBXN8	UBX domain protein 8
CLU	Clusterin	VDAC3	Voltage-dependent anion channel 3
KYNU	Kynureninase	TMEM234	Transmembrane protein 234
HNMT	Histamine N-methyltransferase	ZNF302	Zinc finger protein 302
RIMS2	Regulating synaptic membrane exocytosis 2	GPRC5C	G protein-coupled receptor, class C, group 5, member C
TTLL7	Tubulin tyrosine ligase-like family member 7	PTCD2	Pentatricopeptide repeat domain 2
ZNF141	Zinc finger protein 141	TSTD1	Thiosulfate sulfurtransferase (rhodanese)-like domain-containing 1
SPTLC3	Serine palmitoyltransferase, long chain base subunit 3	C14orf105	Chromosome 14 open reading frame 105
GLMP	Glycosylated lysosomal membrane protein	RAPGEF5	Rap guanine nucleotide exchange factor 5
TLR6	Toll-like receptor 6	MGST2	Microsomal glutathione-S-transferase 2
TPK1	Thiamin pyrophosphokinase 1	MAK16	MAK16 homolog
SERPINB2	Serpin peptidase inhibitor, clade B (ovalbumin), member 2	PLAA	Phospholipase A2-activating protein
ZNF334	Zinc finger protein 334	L3MBTL3	L(3)mbt-like 3 (drosophila)
SERPINB8	Serpin peptidase inhibitor, clade B (ovalbumin), member 8	CADM1	Cell adhesion molecule 1
GRAMD1B	GRAM domain-containing 1B	THEM4	Thioesterase superfamily member 4
ZNF790	Zinc finger protein 790	IL36G	Interleukin 36, gamma
AASS	Aminoadipate-semialdehyde synthase	TNFAIP6	Tumor necrosis factor, alpha-induced protein 6
C12orf60	Chromosome 12 open reading frame 60	WLS	Wntless Wnt ligand secretion mediator
SLCO1B3	Solute carrier organic anion transporter family, member 1B3	GJA1	Gap junction protein alpha 1

Looking at the subcellular location of the differentially expressed genes, we observed that upregulated genes in HLA-G-expressing cells compared to control GFP cells were mostly located in membranes and vesicles (Fig. 2c), while downregulated genes were mostly associated with cytosol (Fig. 2d). As for the molecular functions, those of HLA-G upregulated genes are much diversified, whereas downregulated genes are mostly related to mitochondria activity (Table 2).

**Table 2:** Genes related to mitochondria activity whose expression levels are reduced in HLA-G-expressing cells

Symbol	Gene name	*p* value
CLU	Clusterin	0,00472043
CSF3	Colony stimulating factor 3	6,32938E-05
DSC2	Desmocollin 2	0,000701977
GLMP	Glycosylated lysosomal membrane protein	0,00799334
GLUL	Glutamine synthetase	0,0013335
GSTM3	Glutathione-S-transferase mu 3	0,000173553
MGST2	Microsomal glutathione-S-transferase 2	0,0454994
NSUN3	NOP2/Sun RNA methyltransferase 3	0,00305772
PTCD2	Pentatricopeptide repeat domain 2	0,0495411
TSTD1	Thiosulfate sulfurtransferase-like domain-containing 1	0,0437696
TTLL7	Tubulin tyrosine ligase-like 7	0,00773265
VDAC3	Voltage-dependent anion channel 3	0,0313847
ZNF302	Zinc finger protein 302	0,0359886
ZNF790	Zinc finger protein 790	0,0188719

### Validation of gene expression modifications triggered by HLA-G neo-expression

Before proceeding further with a more thorough analysis of the effect of HLA-G in tumor cells, we performed an expression validation of the transcriptome results by RT-PCR. We confine our analysis to selected genes according to their implication in different biological pathways. These were cell–cell or cell–matrix interactions (ADAM-12), K-voltage channel gated (KCNIP1, KCNMA1), angiogenesis (VEGF-C, NRP1), neural genes (CDR1), cell adhesion (NCAM1), mitochondria dynamics (CLU), bone morphogenetic protein antagonists (GREM1) and TBX3, the only transcription factor found modulated by HLA-G. Specific primers are listed in supplementary Table S1.

**Fig. 3:**
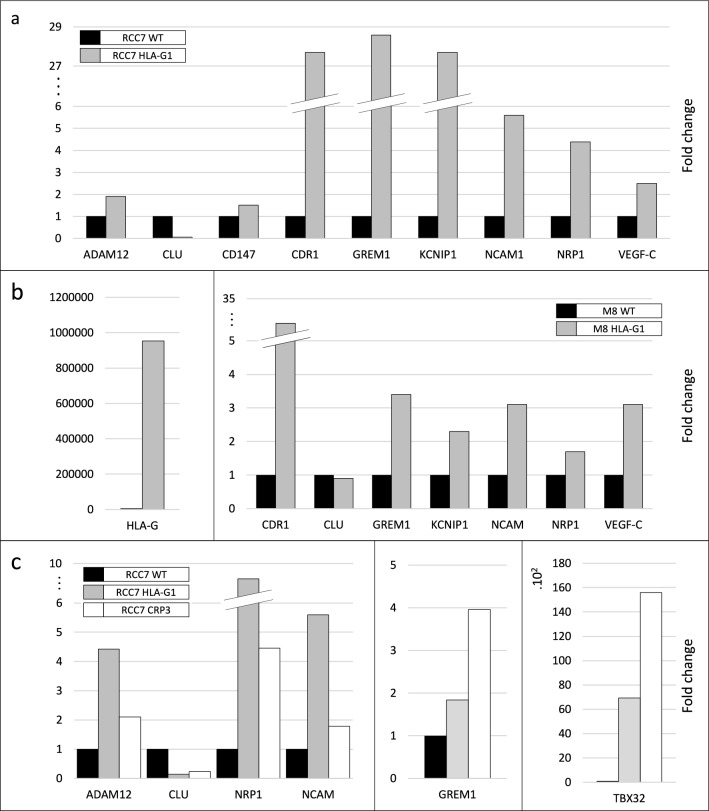
RT-PCR results showing expression modifications of selected genes belonging to different biological pathways observed in HLA-G versus GFP-expressing cells. **a** Expression modifications found in the renal RCC7 tumor line. **b** Gene expression modifications observed in the melanoma cell M8. Left: HLA-G expression in WT and HLA-G transduced cells; right: expression modifications of selected genes belonging to different biological pathways modified by HLA-G. **c** RT-PCR results of the CRISPR/Cas9-mediated edition of HLA-G

The results of the RT-PCR analysis, represented in Fig. 3a, were highly consistent with those obtained with microarray technology. This successfully validates the results of the microarray analysis showing that the neo-expression of HLA-G in RCC7 tumor cells simultaneously modifies genes involved in different biological pathways.

### Impact of HLA-G expression in melanoma cells

To determine whether the molecular modifications of gene expression triggered by HLA-G are cell-dependent, we selected several genes (NRP1, NCAM1, KCNIP1, GREM1, CDR1 and CLU) found dysregulated in the RCC7 tumor cells and measured their expression levels in the melanoma cell line M8, expressing or not high levels of HLA-G (Fig. 3b, left). We select this melanoma cell line since it was in this cell line that was demonstrated for the first time the role of HLA-G in tumor progression [11]. The results demonstrate that the expression levels of the selected genes follow the same trend in melanoma cells than in RCC7 cells (Fig. 3b, right), providing evidence that the effect observed in RCC7 cells is not restricted to this cell type and can be observed in cell lines of different origin. Undoubtedly, future studies carried out in a larger panel of cell lines and tumors would make it possible to confirm the specific core network triggered by HLA-G.

### CRISPR/Cas9-mediated HLA-G knockout validates the direct implication of HLA-G in the expression of several cancer genes

To further study the implication of HLA-G in the gene expression modifications highlighted with the microarray technology, we use the CRISPR/Cas9 gene editing method to knockout HLA-G expression (extensively explained in [10]). We selected four clones in which the disruption of HLA-G protein was confirmed by flow cytometry. These clones displayed a 90–99.3% reduction of HLA-G protein levels. Expression levels of six genes were then measured in these edited cells and compared to parent HLA-G-expressing cells by RT-PCR. An example of a clone with 96.3% HLA-G protein inhibition is shown (Fig. 3c).

The results revealed that the expression levels of ADAM-12, NCAM1 and NRP1 that were increased in the presence of HLA-G were now reduced in the edited cells. Similarly, the levels of CLU, which were reduced following the expression of HLA-G in the cancer cells, were found increased within the CRISPR/Cas9-mediated edited cells (Fig. 3c, left). Unexpectedly, the HLA-G edition led to an increase in GREM1 and TBX32 expression levels (Fig. 3c, middle and right, respectively). The hypotheses put forward to explain this effect are further commented in the discussion section.

### HLA-G is involved in Ca^2+^ signaling

To further deepen the understanding of the effect of HLA-G in tumor cells, we carried forward the dissection of the system using inhibitors of specific molecules. Since our microarray analysis indicated that the expression of several genes related to K^+^/Ca^2+^ channels was highly modified in the presence of HLA-G, we ought to first measure Ca^2+^ release in RCC7 cells expressing or not HLA-G.

**Fig. 4:**
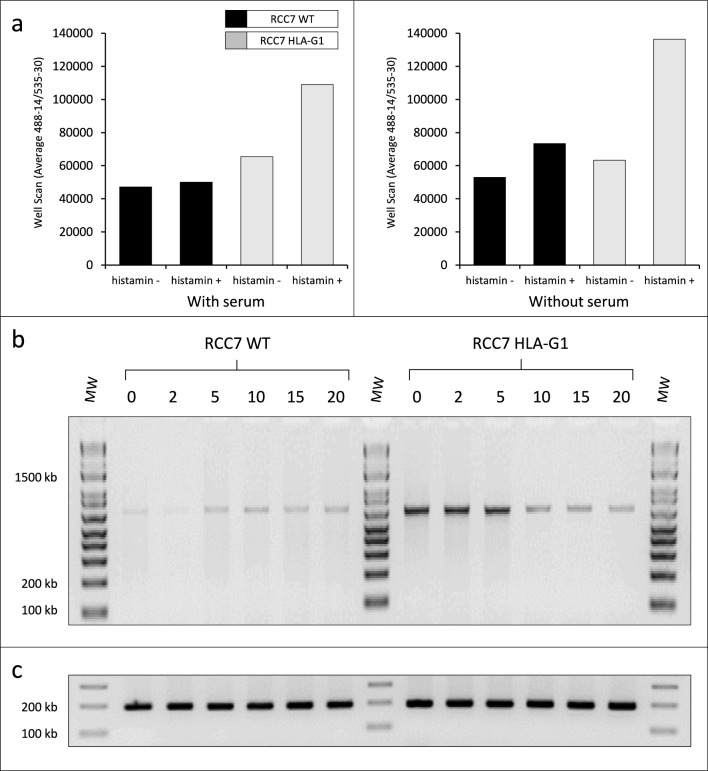
Calcium flux assays. **a** Calcium release in WT versus HLA-G-expressing cells was performed in the presence or not of histamine, in media containing (left) or not (right) serum. The fluorescence intensity was measured at Ex/Em = 490/525 nm. **b** RT-PCR analysis of WT cells (left) and HLA-G-expressing cells (right) following exposure to 0–20 µM of the tBHQ inhibitor using KCNIP1-specific primers. **c** The cDNAs are the same as in (b), amplified with actin primers to verify the integrity of the RNA samples

To this end, 25.000 RCC7 cells (WT and HLA-G1) were settled per well in a Flat-Bottom Plate containing 100 µl of DMEM and 200 mM L-glutamine for 24 h in the presence or absence of serum. Extemporaneously, histamine dihydrochloride was added at a final concentration of 1 µM to induce Ca^2+^ release. The results clearly showed that the Ca^2+^ release efficiency following histamine addition was higher in RCC7-HLA-G1 cells compared with RCC7-WT cells in which the levels of Ca^2+^ remained mainly similar before and after the addition of the calcium activator histamine dihydrochloride (Fig. 4a). The Ca^2+^ release efficiency was not found modified in the presence or absence of serum.

To further highlight the relationship between Ca^2+^ and HLA-G, we cultured cells expressing or not HLA-G in the presence of the calcium flux inhibitor 2,5-t-butylhydroquinone (tBHQ). Cells were grown to confluence and exposed to increasing concentration (2, 5, 10, 15 and 20 µM) of the tBHQ inhibitor for 30 min. RNA was extracted, and the derived cDNAs were analyzed by RT-PCR. We used as paradigm the voltage-dependent K^+^/Ca^2+^ channels (KCNIP1) since the induction ratio of this gene was the highest in this category. Figure 4b shows the results of the RT-PCR analysis using KCNIP1-specific primers. The addition of increasing amounts of tBHQ significantly reduced the expression of KCNIP1 in RCC7-HLA-G1 cells (Fig. 4b, right), whereas measurements of KCNIP1 levels were only slightly modified in RCC7-WT cells (Fig. 4b, left). Moreover, the dose-dependent inhibition of KCNIP1 levels by the tBHQ in the presence of HLA-G is consistent with the upregulation of KCNIP1 levels by HLA-G found with microarrays.

### The expression of HLA-G is related to the angiogenic receptor NRP1

**Fig. 5:**
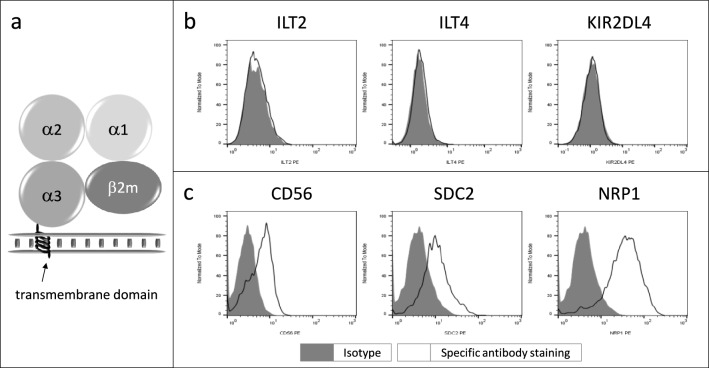
Expression analysis of common and putative HLA-G receptors. **a** Schematic representation of the structure of HLA-G, emphasizing its small cytoplasmic domain, **b** flow cytometry analysis of the most known HLA-G receptors, and **c** novel receptors found overexpressed in HLA-G-expressing cells compared to WT cells

We then ought to identify putative receptor(s) that might be responsible for the transmission of so many different biological signals triggered by the presence of HLA-G in tumor cells since such broad biological effects may not be only attributable to the so small cytoplasmic domain of HLA-G diagrammatically represented in Fig. 5a.

We searched first for the presence, in the RCC7 cell line, of the most well-known HLA-G receptors ILT2, ILT4 and KIR2DL4 by flow cytometry using specific antibodies. The results showed that the expression of these receptors was undetectable by this technique (Fig. 5b). Therefore, we further looked for the presence of other receptors that could be partners of HLA-G in these cells. We tested CD56, SDC2 and NRP1 whose transcript levels were increased in the presence of HLA-G. The flow cytometry analysis of RCC7 cells confirmed the presence of these receptors in RCC7 cells (Fig. 5c). This could suggest that these molecules might be considered as potential HLA-G partners.

**Fig. 6:**
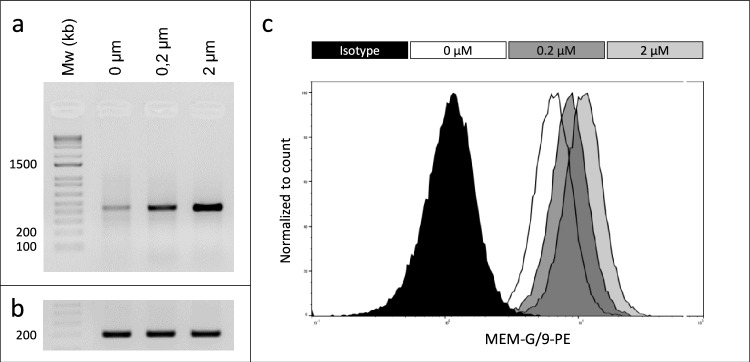
Relationship between NRP1 and HLA-G. RCC7-HLA-G cells were grown to confluence and exposed to increasing concentrations (0, 0.2 and 2 µM) of the EGO1377 inhibitor for 30 min. **a** Results of the RT-PCR analysis using HLA-G-specific primers. **b** The cDNAs are the same as in (a), amplified with actin primers to verify the integrity of the RNA samples. **c** Flow cytometry analysis using the HLA-G-specific antibody MEM-G/9-PE

We therefore oriented our study toward the search of a putative relationship between these receptors and HLA-G. We mainly choose NRP1, given the involvement of this receptor in angiogenesis and the entry of the SARS-CoV-2, which both were found to be related to HLA-G. To this end, cells expressing or not HLA-G were treated with the NRP1 inhibitor EGO1377. Cells were grown to confluence, exposed to increasing concentrations (0, 0.2 and 2 µM) of the EGO1377 inhibitor for 30 min and divided in two equal parts for the RNA and protein analysis. Following the extraction of RNA, the derived cDNAs were analyzed by RT-PCR using specific primers. Unexpectedly, the results of the RT-PCR analysis showed that the only gene tested whose expression was modified in the presence of the inhibitor EGO1377 was HLA-G. Increasing the concentration of EGO1377 produced an increase in HLA-G transcript levels (Fig. 6a). In parallel, the cells exposed to different concentrations of the inhibitor EGO1377 were analyzed by flow cytometry using the HLA-G-specific antibody MEM-G/9. Similarly, the results demonstrate an increase in the HLA-G protein levels using this method (Fig. 6b). This adds to the literature reports showing the biological effects of this inhibitor on VEGF/NRP1 (EGO1377)/SARS-CoV-2, which, at present, may relate them to HLA-G.

## Discussion

Compelling evidence supports the role of HLA-G in immune tolerance, viral infection, autoimmune diseases and protection of transplanted tissues, via the inhibition of all immune cell effectors (T, B, NK and APC). However, data concerning the consequence of HLA-G expression in tumor cells are far from being available even though high incidence of HLA-G expression has been reported in several tumors such as ccRCC. In particular, the molecular pathways triggered by the presence of HLA-G are mostly unreported.

Here, to unravel potential roles of HLA-G in cancer cells, the RCC7 cell line derived from a ccRCC patient was used as model. We compared the transcriptome of these tumor cells with those in which we induce the expression of HLA-G. The data pinpoint, for the first time, a number of key pathways modified by the presence of HLA-G and illustrate the complexity of the biological networks triggered by this immune checkpoint (Figure S1). Of the 133 most misregulated genes found, the upregulated genes encode proteins that belong to diverse categories, essentially present at the membrane. The top-ranking genes encode ion channels and proteins involved in angiogenesis and tumor development. In this context, it is worth mentioning that several expression networks found in our study correlate with those previously reported in the literature (see below). This, and the validation of the microarrays results through different techniques such as RT-PCR, protein analysis and HLA-G edition using the CRISPR/Cas9 method, adds to the pertinence of our findings.

Notably, following HLA-G neo-expression, the downregulated genes belong to less diverse categories than upregulated genes. They are mainly found in the cytoplasm, related to mitochondria dynamics. In fact, emerging evidence has demonstrated that modified dynamics of mitochondria and, particularly, the regulation of intracellular messengers such as Ca^2+^ by transport channels correlate with pathological states [12, 13] and constitute an important hallmark of ccRCC. Here, we show, for the first time, that HLA-G downregulates the expression of genes included in this category.

Mitochondria are often considered the cell’s powerhouse, for their essential role in the production of adenosine triphosphate (ATP) through oxidative phosphorylation and other biochemical pathways such as pyrimidine and purine biosynthesis. Damage to mitochondria results in a progressive respiratory chain dysfunction that might lead to various diseases including cancer. The mitochondria damage might be prevented by antioxidants such as superoxide dismutase 2 (SOD2), catalase, glutaredoxin 2 (GLRX2), reduced glutathione (GSH), glutathione peroxidase (GPX) and thioredoxin 2 (TXN2). The absence of these antioxidants impairs, therefore, mitochondria protection. Notably, the levels of expression of these genes, added to six other genes involved in mitochondria activity (TST, NSUN3, GLMP and PTCD2), were reduced following the expression of HLA-G in the RCC7 cells (Table 2), consistent with mitochondrial impairment found in many types of cancer, particularly in ccRCC.

The reduced levels of expression of other genes such as clusterin (CLU), a stress-responding protein associated with cytoprotection, also aggravate mitochondria dynamics. Moreover, CLU is involved in the biological effects of Ca^2+^. Likewise, impact of Ca^2+^ homeostasis regulates CLU gene activity and transcription. In addition, reduced expression of desmocollin 2 (DSC2), a calcium-dependent cell–cell adhesion transmembrane glycoprotein, favors tumor progression and poor survival through redistributing adherent junctions [14]. The extremely high reduction in CLU and DSC2 levels found in our microarray study following HLA-G expression is consistent with HLA-G-favoring tumor progression.

Increasing evidence during the past decade indicates that different ion channels are expressed in several cancers in humans and regulate a multitude of cellular processes, including migration, invasion and proliferation. Of the channels considered important for cancer progression, special interest has been given to Ca^2+^ channels. Here, we show the relationship of HLA-G with Ca^2+^ hemostasis, based on the large number of genes involved in Ca^2+^ metabolism such as KCNIP1, KCNMA1 and TRPC4. Interestingly, these genes were previously reported to act in concert in other systems [15–17]. In addition, we show that the calcium flux inhibitor 2,5-t-butylhydroquinone tBHQ [18] reduces the expression of KCNIP1 only in the presence of HLA-G. Notably, these ion channels were also found to contribute to regulate the intracellular Ca^2+^ influx in angiogenic process and tumor progression [19].

The relationship of Ca^2+^ influx and angiogenesis has been visible in our study by the large number of genes belonging to this category that were found modified by the presence of HLA-G. In particular, VEGF-C, widely recognized as one of the driving forces for tumor progression and invasion, was previously described as a target of HLA-G [20] and acts directly on endothelial cells to induce Ca^2+^ signaling [21]. Among other co-enriched genes involved in angiogenic processes and modulated by HLA-G, we found ADAM-12, BAG1, CHRDL1, CLU, GREM1, ITGA2, MYOF, NCAM1, NRP1, SDC2, TGFB2 and VCAN.

ADAM-12, a disintegrin and metalloproteinase domain-containing protein, is significantly positively correlated with the key angiogenic regulator VEGF. ADAM-12 is a novel regulator of tumor angiogenesis found upregulated in epithelial cancers [22, 23]. The pro-tumorigenic effects that it exerts are independent of its own protease function and contribute to increase tumor proliferation, metastasis and tumor angiogenesis.

ADAM-12 is a target of TBX3, the only transcription factor, found highly upregulated following the neo-expression of HLA-G. The levels of TBX3 in RCC7 cells are extremely low, but they were significantly upregulated in response to HLA-G. Genes encoding transcription factors act on a great number of molecules simultaneously and are often deregulated in cancer cells. It has been estimated that transcription factors account for 20% of all oncogenes identified so far [24, 25]. At present, no known function was attributed to TBX3 but is frequently overexpressed in a wide range of epithelial and mesenchymal-derived cancers [26]. TBX3 shares the top-ranking positions of transcription factors (14 cohorts) that promotes cell migration and invasion. Elevated TBX3 mRNA levels strongly correlated with (ER)-positive breast cancer tumors [27]. The literature reports also demonstrate that TBX3 upregulates important gene targets involved in tumor development, angiogenesis and ion transport such as versican (VCAN), CD9, chromo-domain helicase DNA binding protein 1 (CHDR1) and CD86. Notably, these target genes were also found upregulated by HLA-G in our microarray survey. These results would be consistent with HLA-G triggering a large number of molecular cascades through TBX3 which in turn would result in worsening tumor aggressiveness.

Neuropilin-1 (NRP1), a co-receptor of VEGF, was also found upregulated by HLA-G in this study. It is a transmembrane glycoprotein molecule that plays key roles in various biological processes, including neuronal development, angiogenesis, vascular permeability, immune functions and tumor angiogenesis, and enhances the proliferation of tumor cells and metastasis [28, 29]. NRP1 lacks direct signaling capabilities and, therefore, acts as a co-receptor that binds a signaling molecule in addition to the primary receptor, thereby affecting ligand–receptor activity. The HLA-G-mediated modifications of the expression of these two receptors are consistent with previous hypothesis suggesting that NRP1 and VEGFR 1/2 may be co-regulated by some upstream molecular mechanism [28].

In some cases, it would be essential to block NRP1, as in the case of the coronavirus-induced disease 2019 (COVID-19) triggered by the severe acute respiratory syndrome coronavirus 2 (SARS-CoV-2) which is the causative agent [30]. This coronavirus can use NRP1 to enter cells through the spike protein and enhance the entry and infectivity of SARS-CoV-2 [31] since it is, as angiotensin-converting enzyme 2 (ACE-2), an entry point of the virus [32]. Disrupting the key protein–protein interactions between NRP1 and VEGF-A at the virus binding site might reduce viral infectivity. In this context, small-molecule NRP1 antagonists or inhibiters such as the inhibitor EGO1377 have been developed for their ability to successfully interfere with the VEGF-A binding and prevent the SARS-CoV-2 viral entry [33]. It is interesting to note that the NRP1 inhibitor EG01377, against all odds, raises the expression of HLA-G at the transcript and protein levels. This demonstrates a strong relationship between NRP1 and HLA-G.

We might even hypothesize that HLA-G and NRP1 are physically linked. And then, when the inhibitor EGO1377 is added, it will cause a change in the conformation of NRP1 that prevents the link with HLA-G. Therefore, HLA-G regains its usual conformation and can again be recognized by the HLA-G-specific antibody MEM-G/9 which results in an increase in HLA-G expression when analyzed by flow cytometry. Moreover, HLA-G has recently been shown to be related to the SARS-CoV-2 virus [34–36]. Similarly, the extracellular matrix metalloproteinase inducer (EMMPRIN), also known as cluster of differentiation 147 (CD147) or BASIGIN, found upregulated by HLA-G in this study, has also been reported as enabling the SARS-CoV-2 virus entry and replication within host cells [37]. In particular, the CD147 rs8259T > A single nucleotide variant on SARS-CoV-2 susceptibility is a major risk factor for COVID-19 [38].

CD147 is also emerging as a protein capable of regulating cancer hallmarks [39], such as tumor angiogenesis, a process in which CD147 acts through the regulation of VEGF/VEGFR [40] and ADAM-12 [41].

The relationship between CD147 and HLA-G is seen in cancer cells where CD147 is emerging as a protein capable of regulating cancer hallmarks [39], such as tumor angiogenesis, a process in which CD147 acts through the regulation of VEGF/VEGFR [40] and ADAM-12 [41]. Moreover, at the level of tissue transplantation, HLA-G and CD147 blockade would be very important to avoid graft rejection [42].

Pre-eclampsia, whose pathophysiology and etiology remain mostly undefined, represents a leading consequence of fetal and maternal mortality and morbidity. Pre-eclampsia placentas have been correlated with the downregulation of HLA-G. Notably, several independent studies reported that genes that were found modulated by HLA-G in this study such as VEGF, NRP1, NCAM1 [43], clusterin and glutathione-S-transferase [44] are also related to pre-eclampsia. Even though the functional relevance of these correlations is yet unknown, it shows once again the close association of this gene network with HLA-G.

The specific implication of HLA-G in the expression of NRP1 as well as other genes such as NCAM1 was further demonstrated by the edition of HLA-G from RCC7 cells using the CRISPR/Cas9 gene editing method. Indeed, the use of this method allowed reversing the induction mediated by HLA-G in these cancer cells. In some cases, however, the inhibition of HLA-G following CRISPR/Cas9 edition could not reverse the initial upregulation, and even more surprisingly, the expression levels were higher than those initially found. This phenomenon was also demonstrated in other systems such as in GREM-CRISPR/Cas9-silenced cells, another gene found upregulated by HLA-G in this study. GREM1 expression is associated with collagen formation, angiogenesis and extracellular matrix (ECM) organization. It is frequently overexpressed in several cancers and correlates with poor prognosis [45]. GREM-CRISPR/Cas9-silenced cells do not behave in the same manner [46]. For example, some clones formed smaller primary tumors and, among other features, loose their ability to metastasize to the lungs. Alternatively, the unexpected increase in the expression of some genes following HLA-G editing might be consistent with the prolonged and irreversible expression of HLA-G target genes that in turn will induce the irreversible activation of signaling pathways and networks that will persist in the tumor cell even in the absence of HLA-G. This long-lasting effect was also found in other systems [47].

Consistent with the transcriptomic findings, flow cytometry analysis revealed a greater expression of the neural cell adhesion molecule NCAM 1 (CD56) in cells expressing HLA-G compared to WT cells. CD56 is an immunoglobulin superfamily cell adhesion molecule known to be expressed physiologically on normal tissues such as natural killer cells (NK cells), neuroendocrine glands, the central and peripheral nervous system and cardiomyocytes. At present, NCAM1 is consider not only as a simple molecular anchor enabling mechanical cell adhesion but rather an important functional receptor mediating intracellular downstream signaling associated with aggressive biological behavior, increased metastatic capacity, tumor angiogenesis and poor prognosis [48].

Here, we demonstrate, for the first time, that de novo HLA-G expression is consistent with an increase in NCAM1 transcript and protein levels. The use of the CRISPR/Cas9 method to edit HLA-G from these cancer cells has indeed succeeded in reversing the induction mediated by HLA-G in wild-type RCC7 cells, demonstrating the direct implication of HLA-G in the regulation of NCAM1 expression.

Taken together, the overall picture emerging from this study is that HLA-G has complex roles in tumor cells than initially anticipated. This is consistent with recent reports showing that genes such as ADAM-12 or NCAM1, beyond their known function, have novel and unexpected roles in a variety of biological pathways such as tumors progression.

Since there is no known HLA-G receptor (such as ILT2, ILT4 or KIR2DL4) present on the tumor cells used in this study and given the large number of interacting physiological ligands and receptors that can come into play in a cell [49], it might be conceivable that these cells possess not yet characterized receptors, that might act in concert with HLA-G responsible for the modified tumor response.

Altogether, the data generated in this study provided a more comprehensive understanding of the complex mechanisms behind the cross-talk between tumor and immune cells and highlighted, for the first time, potential HLA-G partners. These associations orchestrate a more complex than initially anticipated interplay of biological pathways and give to HLA-G potentially new pathophysiological properties that, in turn, have to be studied in a variety of tumors of different origin. Deepening our knowledge on the way in which genes and proteins interact to form these networks would certainly lead to the design of improved therapeutic applications.

## Supplementary Information

Below is the link to the electronic supplementary material.

Supplementary file1 (DOCX 624 kb)

Supplementary file2 (DOCX 60 kb)

## Data Availability

The datasets generated and/or analyzed during the current study are not publicly available, since no repository exists, but are available from the corresponding authors.

## Abbreviations

ADAM-12: A disintegrin and metalloproteinase domain-containing protein 12
BAG1: BCL2-associated athanogen
ccRCC: Clear cell renal cell carcinoma
CDR1: Cerebellar degeneration-related 1
CEA: Atomic Energy and Alternative Energies Agency
CHRDL1: Chordin-like 1 gene
CLU: Clusterin
CRISPR/Cas9: Clustered regularly interspaced short palindromic repeat and associated protein Cas9
DSC2: Desmocollin
GFP: Green fluorescent protein
GREM1: Gremlin 1 gene
HLA-G: Human leukocyte antigen G
IC: Immune checkpoints
ILT2: Ig-like transcript 2 receptor (LILRB1: leukocyte immunoglobulin-like receptor B1)
ILT4: Ig-like transcript 4 receptor (LILRB2: leukocyte immunoglobulin-like receptor B2)
ITGA2: Integrin subunit alpha 2
KCNIP1: Potassium voltage-gated channel interacting protein 1
KCNMA1: Potassium calcium-activated channel subfamily M alpha 1
KIR2DL4: Killer cell immunoglobulin-like receptor, two Ig domains and long cytoplasmic tail 4
MYOF: Myoferlin
NCAM1: Neural cell adhesion molecule 1 (CD56)
NRP1: Neuropilin-1
RT-PCR: Reverse transcriptase-polymerase chain reaction
SDC2: Syndecan 2
tBHQ: Calcium flux inhibitor, 2,5-t-butylhydroquinone
TGFB2: Transforming growth factor beta 2
TRP4: Transient receptor potential cation channel, subfamily C, member 4
VCAN: Versican
VEGF: Vascular endothelial growth factor
