# Supplementary material for: HLA-G neo-expression modifies genetic programs governing tumor cell lines
Source: Cancer Immunol Immunother. 2024 Oct 3;73(12):247. doi: 10.1007/s00262-024-03768-5 (PMC11447172; doi:10.1007/s00262-024-03768-5)
Supplement: Supplementary file 1 [file 262_2024_3768_MOESM1_ESM.docx]

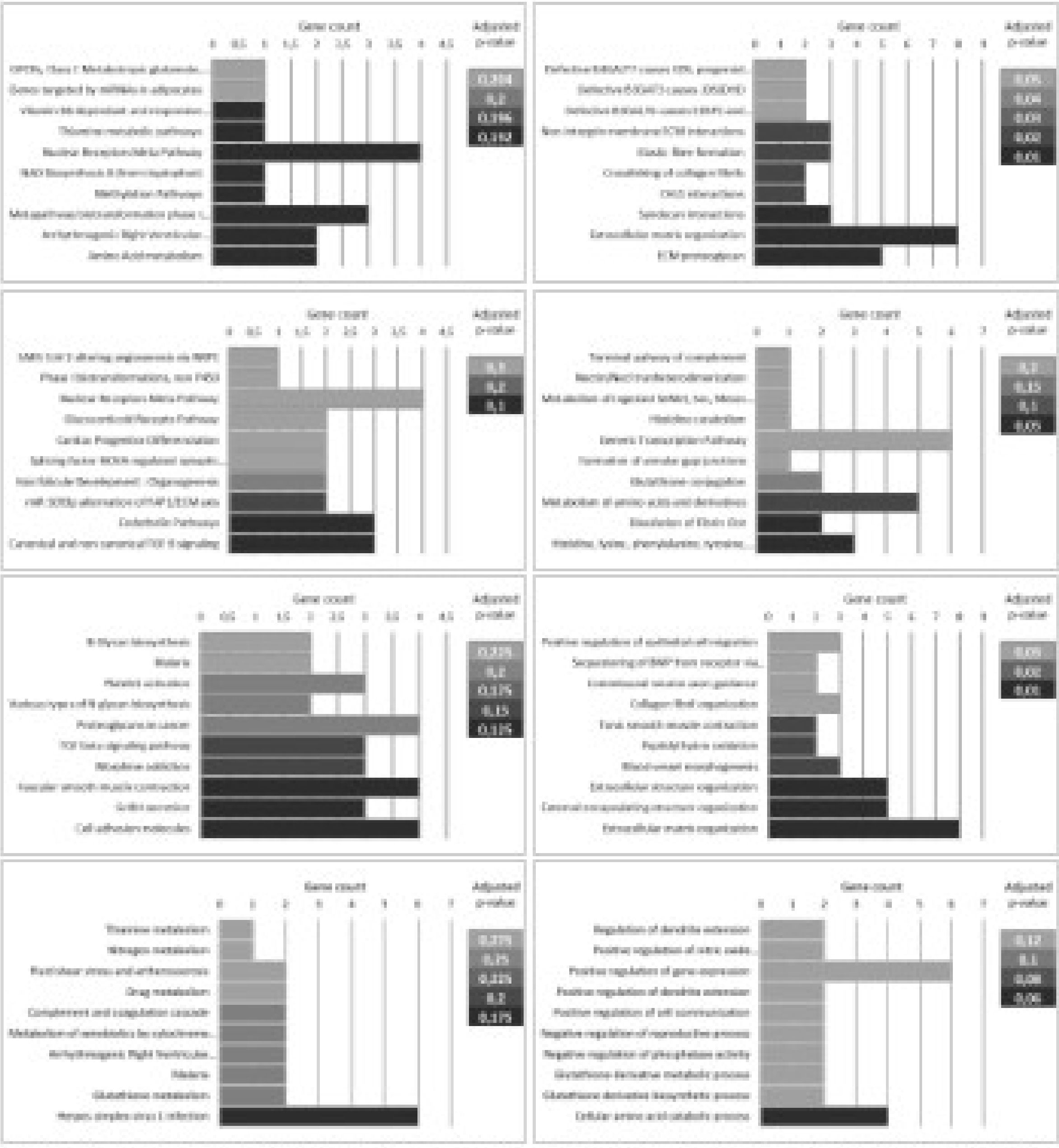

Figure S1: Biological process enriched in the annotations of differentially expressed genes between HLA-G and GFP expressing RCC7 cells.
