# Supplementary material for: HLA-G neo-expression modifies genetic programs governing tumor cell lines
Source: Cancer Immunol Immunother. 2024 Oct 3;73(12):247. doi: 10.1007/s00262-024-03768-5 (PMC11447172; doi:10.1007/s00262-024-03768-5)
Supplement: Supplementary file 2 [file 262_2024_3768_MOESM2_ESM.docx]

PRIMERS	SEQUENCES
ADAM12.F<br>ADAM12.R	TCGACTACAACGGGAAAGCA<br>ACACGTGCTGAGACTGACTG
CDR1.F<br>CDR1.R	ACACAAGTAGGCTGGAAGACATTA<br>AATCCACATCTTCCAATGGCCT
CLU.F<br>CLU.R	TGCCTGAAACAGACCTGCAT<br>CATGACATCAGCATGTGCG
GREM1.F<br>GREM1.R	GGAGCCCTGCATGTGACG<br>GAAGCGGTTGATGATGGTGC
HLA-G.F<br>HLA-G.R	GGAAGAGGAGACACGGAACA<br>CCTTTTGTTCAGCCACATTGG
KCNIP1.F<br>KCNIP1.R	TGAGCTGGAGATGACCATGG<br>GGCAGATCAGGGTGTTAAGG
KCNMA.F<br>KCNMA.R	TGGCCTCCTCCATGGTGA<br>TTCTGGGGCCTCCTTCGTCT
NCAM1.F<br>NCAM1.R	TACACTTGGGAGGGGAACCA<br>CTCCACCTGGTCGATGGATG
NRP1.F<br>NRP1.R	GAAGCACCGAGAGAACAAGG<br>CTCGGGGTAGATCCTGATGA
TBX3.F<br>TBX3.R	TGTACATTCACCCGGACAGC<br>CTGGTATGCAGTCACAGCGA
VEGFC.F<br>VEGFC.R	ATGTGTGTCCGTCTACAGATGT<br>GGAAGTGTGATTGGCAAAACTGA

Table S1: Primer sequences of genes used in RT-PCR analysis.
